# Improving Clinical Practice: What Dentists Need to Know about the Association between Dental Fear and a History of Sexual Violence Victimisation

**DOI:** 10.1155/2015/452814

**Published:** 2015-01-12

**Authors:** Houman Hadad Larijani, Marika Guggisberg

**Affiliations:** ^1^Pacific Smile Group, Melbourne, VIC 3000, Australia; ^2^Curtin University, Perth, WA 6845, Australia

## Abstract

Anecdotal evidence suggests lack of dentist knowledge and uncertainty about how clinical practice can be improved when dealing with victims of sexual violence. This systematic review presents a synthesis of the available literature, which examined the association between dental fear and a history of sexual violence victimisation. All studies indicated, to various degrees, that dental fear is associated with a history of sexual violence victimisation. The analysis identified several common themes including a perception of lack of control, avoidance behaviours, experiences of flashbacks, feelings of embarrassment, difficulties with the physical proximity to the dentist, the sex of the dentist reminding patients of the perpetrator, being placed into a horizontal body position, the specific impact of fellatio, the smell of latex, experienced lack of knowledge of dental professionals leading to insensitive treatment as well as revictimisation experiences, and the occurrence of disproportionate dental problems among patients who had experienced event(s) of sexual violence. All these themes are discussed in detail. Specific strategies are offered to assist dental practitioners in providing sensitive treatment for patients with a history of sexual violence. Additionally, several suggestions are made that may assist both researchers and dental practitioners alike.

## 1. Introduction

Dental fear has been recognised as an important clinical problem hampering the provision of oral health care [1]. Despite improvement of the painless dentistry and increased awareness of dental practitioners that building trusting relationships is crucial, dental fear still remains a challenging issue for both dentists and patients [2]. The concept of “dental fear” refers to patients' feelings of anxiousness and worry about dentistry and receiving future dental care. This fear can range from mild anxiety to extreme fear [1]. The experience of dental fear was identified as an important barrier to dental health care, which can have long-term consequences [3, 4].

Dental patients suffering from dental fear experience higher levels of psychological distress related to dental treatment than those with no fear [5]. Extreme dental fear is associated with poor oral health [1, 6, 7], as a result of less frequent or irregular dental visits, cancelation, and/or deferrals of appointment and treatment [3, 4]. Dental fear has also been associated with increased oral ill health, impaired self-rated oral health, social withdrawal, and an eventually greater need for dental treatments [8].

An estimated 8–19% Australians experience dental fear [9], in spite of changes in teaching and practice of dentistry. The majority of Australians experience various levels of dental fear, but a small significant number of patients suffer from a severe form with a significant impact [10]. High levels of dental fear are associated with poorer oral health outcomes such as decayed and missing teeth [1].

Adults with a history of sexual violence victimisation may experience particular difficulties visiting the dentist due to high levels of fear [11]. Generally, sexual violence is experienced by both genders in all communities, cultures, religious and ethnic backgrounds, and economic and social classes [12]. However, sexual violence is a gendered crime with females usually being victims, while males almost always are perpetrators [13, 14].

Victims of sexual violence may find it especially problematic to tolerate dental treatments and frequently cancel scheduled dental appointments or avoid dental treatment [15]. Researchers found that visiting a dentist for dental treatment can elicit unwanted memories [11, 16]. This may result in experiences of disempowerment and anticipation of pain and/or discomfort [16].

Perpetrators frequently groom vulnerable individuals by starting with activities that appear safe. They often attribute positive motives to their sexual behaviours, which suggest love or necessary education [11]. In a dental setting, patients are asked to trust the dentist, who frequently assures patients that even though they may experience painful or unpleasant situations during the treatment, it will be beneficial for them in the end [15]. This may be a similar experience to the victimisation event(s) and can lead to perceived revictimisation or fear thereof.

Some characteristics of patients who had experienced sexual violence in dental surgery include dislike of being touched, fear of judgment and sensitivity to perceived criticism, sensitivity to particular smells, and especially sensitivity to having instruments in the mouth [16, 17, 19]. Since sexual violence may involve the victim's mouth, oral and dental treatment may be particularly challenging, even threatening, given the requirement of the patient's mouth being touched and intruded by the dentist's fingers and instruments [11]. Consequently, destructive psychological impacts of sexual violence may negatively affect the ability to enjoy benefits of dental care [12].

### 1.1. General Health Effects of Experiencing Sexual Violence

Sexual violence is identified as a serious public health problem [13, 25]. A significant body of research found a strong association between sexual violence victimisation and poor health outcomes [13, 14, 25, 26]. It can affect victims' mental and physical health across the life course [27, 28]. Victims of sexual violence, for instance, have been shown to be at an increased risk for anxiety disorders, depression, posttraumatic stress disorder [13, 28], obesity and eating disorders, suicide and self-harm behaviours [29], and health risk behaviours such as alcohol/other drugs use [14, 30]. Victimisation by sexual violence can significantly influence nearly all aspects of victims' lives including the ability to participate in the workforce and social relationships [13, 31].

In the aftermath, both genders often feel out of control and disempowered as they may realise that their wills and desires do not count [12]. Furthermore, victims may learn not to care about themselves and ignore or disregard generated signals by their bodies such as pain, injury due to the effects of sexual violence [12, 31]. Long-term coping skills may include compulsive behaviours masking their emotions that originated from experiencing sexual violence [12, 13].

### 1.2. Oral Health Effects of Experiencing Sexual Violence

Considering the prevalence of sexual violence, Leeners and colleagues [11] anticipated that nearly 20% of female patients requesting dental care might have experienced sexual violence in the previous stages of their lives. This suggests that dentists may work with the victims of sexual violence on a regular basis without any awareness of their history. Therefore, the clinical implications of managing dental patients with sexual violence victimisation have to be considered.

Some studies indicated that the experience of sexual violence could potentially lead to the development of fear, anxiety, and/or dissociation in victims, resulting in poor relationship between the victims and health care practitioners, particularly the dentist [16–19]. This issue may in turn result in misdiagnosing of existing dental problems as well as considering the victims as difficult dental patients [15]. Additionally, published evidence indicates that sexual violence victims tend to have low self-worth and low self-esteem, which may force them to avoid attending the dental setting for enjoying the dental treatments [32]. Thus, it may not be surprising to observe high rate of oral and dental diseases among those who experienced sexual violence. Dougall and Fiske [12] argued that sexual violence victims are particularly likely to avoid visiting the dentist, which* per se* results in the deterioration of their oral health, and, as a consequence, problems including pain and discomfort.

Although the association between experiencing sexual violence and medical care has been established for some domains of health care such as internal medicine or gynecology and obstetrics [11], knowledge of the association between a history of sexual violence, dental fear, and oral health is in its infancy [16]. Limited publications in this field with gaps in knowledge suggest a need for a systematic review to better establish the association between dental fear and a history of sexual violence victimisation. This paper fills this gap in the literature.

## 2. Methods

A comprehensive literature search identified publications that investigated the association between dental fear and a history of sexual violence victimisation. Different databases including Pubmed, Medline, Google scholar, ProQuest, ScienceDirect, and Willey online were searched using a variety of search strategies. Furthermore, a manual search of key journals was conducted including hand search of hard copies of the Australian Dental Association Journal.

A strict protocol was followed. Original articles included in the systematic review were appraised and synthesized in a rigorous way. The review protocol is outlined below. The primary author screened the abstracts of many studies and was able to reject those papers identified as not fulfilling the inclusion criteria. A long list of papers was created of papers that required to be read in more detail and further studies were located through the reference lists of obtained articles. Data extraction yielded a relatively small number of studies. A record was created for search terms used (Table 1) either individually or in combination and the decision-making process of including or rejecting the paper in the analysis. The critical appraisal of studies was undertaken to assess whether the studies met the inclusion criteria. The second author reviewed and checked the information provided, and consensus method was applied to make the final decision on whether or not to include a study [33]. After all the studies were located and after deciding which ones to include in the review, data were collated and summarized with a descriptive analysis. Key elements of the studies are provided in Table 2 which provides details on study and participant characteristics as well as intervention and results.

### 2.1. Inclusion Criteria

The following three criteria needed to be fulfilled for studies to be included:

- investigation of the association between dental fear and sexual violence (either as an independent event or as a part of a series of variables under investigation);
- studies written in English language;
- studies published between 1995 and 2011.

### 2.2. Data Extraction

The initial search located 18 articles (published between 2000 and 2011). After screening the titles and reviewing the abstracts, 10 studies were excluded, as they did not meet the inclusion criteria. The remaining eight studies were reviewed and critically appraised. Given the limited number of obtained articles it was decided to relax the inclusion criteria and repeat the procedure with a timeframe between 1995 and 2011. Six additional articles were located of which two examined the association between dental fear and a history of sexual violence victimisation. Consequently, 10 articles were included in the final analysis.

### 2.3. Data Synthesis

Obtained information from the studies included in this research was coded. Each publication was allocated a study identification number. The coding scheme collected data on participants' characteristics (e.g., age, gender), year of publication, setting (e.g., community or clinical), location (e.g., country and city), research design (e.g., quantitative, qualitative, or mixed method), variables of interest (e.g., childhood sexual abuse), prevalence (number and percentage) of participants with dental fear, and a history of sexual violence and specific findings (e.g., presence or absence of an association between dental fear and a history of sexual violence victimisation).

## 3. Result and Discussion

Analysed data are presented narratively. It was found that the studies were heterogeneous and it was decided that results should be summarised and presented most appropriately in narrative form. No attempt was made to undertake a statistical synthesis. Themes that emerged from the included publications will be discussed below. First, a brief summary of the included studies will be presented.

### 3.1. Overview of the Included Publications

The following section presents the results of this analysis discussing the main themes that emerged (see Table 3). It provides the findings extracted from the studies included in this review.

### 3.2. Lack of Control (Theme 1)

Lack of control was reported by all the included studies, making it the most common theme. The concept of control, as a main predictor of health behaviour in psychological research [34], is defined as the perception that an individual has the necessary skill, talent, resources, or opportunities to acquire desirable outcomes or avoid harmful situations through his or her own actions [34].

#### 3.2.1. Sexual Victimisation and General Perception of Control

Locus of control (LOC) theory [35] provides a framework in which the impacts of sexual violence victimisation on generalised perception of control can be assessed [36]. It refers to the extent to which individuals believe they can control subsequent events affecting them [35, 37].

Wade and Tavris [38] reported an association between individuals' wellbeing and locus of control, arguing that people with external LOC are more likely to develop psychological disorders than those with higher internal LOC who were found to be less likely to experience physical and psychological symptoms. Similarly, people with high internal LOC were found to have a higher capacity to cope with daily stressors and psychiatric symptoms, which frequently occur in victims of sexual violence [13, 39].

Abramson and Seligman [40] argued that when stress is uncontrollable, individuals experience heightened arousal, which was found to be a key feature of anxiety. In this regard, Hood and Carter [41] reported that individuals with a history of CSA were more likely to develop external LOC given their lack of control over future events.

#### 3.2.2. Concept of Control and Dental Fear

Control as a crucial issue in the development of dental fear was recently examined by Armfield and colleagues [10, 42]. Both studies found that the perception of lack of control was a main concern among dental phobic patients and strongly associated with dental fear. Armfield et al. [42] assessed the association between dental fear and perceived lack of control among 3,937 participants as part of the National Survey of Adult Oral Health (NSAOH), in Australia between 2004 and 2006. The study found that the perceptions of uncontrollability and dangerousness were significantly associated with high levels of dental fear.

In a National Dental Telephone Interview Survey (NDTIS) using 1,084 Australian adults, Armfield [10] examined whether dental fear can be explained by negative dental experiences and/or by cognitive perceptions of going to the dentist. The study found that a perception of uncontrollability was a superior predictor of dental fear compared with negative dental experiences. This finding corroborated previous results indicating a strong relationship between lack of control and dental fear.

#### 3.2.3. Dental Fear, Sexual Violence Experience, and Lack of Control

All studies reported that participants experienced various degrees of helplessness, powerlessness, and incidents of lack of control during dental treatments. The experience of such feelings was believed to be the underlying problem in developing fears of feeling claustrophobic, experiencing chocking, severe gagging or being unable to breathe, and being trapped in the dental chair which interferes with dental treatments [17]. The following statement by a dentist provides evidence of this view: “She reported extreme fear of being trapped in the dental chair—by contrast her fear of needles, drills and painful treatment was low” [17, page 485].

Feelings of helplessness and powerlessness in dental patients with sexual violence histories can potentially result in revictimisation if dental treatment is perceived to be fear provoking [15, 17, 19]. It stands to reason that experiencing such feelings stems from the similarity between sexual violence and perceived lack of control when visiting the dentist. In both situations a perception of victimisation may be evoked through power imbalance and a sense of helplessness and fear.

#### 3.2.4. Strategies to Increase Patient Control

Control is an issue of paramount importance and feeling safe in medical and dental environments is a critical factor for patients with a history of sexual violence [31]. Strategies addressing this issue can significantly help such dental patients to feel safer in the dental environment. Some of the studies reviewed proposed strategies to increase patients' sense of control in dental settings [11, 16, 17]. They are discussed below.

*Collaboration*. Collaboration with patients was identified as one of the most important strategies. Walker et al. [17] argued that particularly people having experienced sexual violence require transparent communication and involvement in treatment decisions to share control. The utilisation of obtaining informed consent before each step of treatment was suggested by several studies [15, 16, 31, 43], involving patients in the treatment procedures. Asking permission before performing dental treatments will give simply back the control to the dental patients with history of sexual violence [12]. 

*“Inform before You Perform.”* The dentist-patient communication was addressed with the following slogan by Stalker et al. [15]: “inform before you perform” (page 1280). The researchers found that pretreatment communication and detailed explanation of the treatment plan were appreciated by the participants and reduced their levels of distress and fear. For example, one participant stated, “I find her [the dentist] good because she does explain everything that she is going to do and why she's doing it at the time and sort of checks, “Is that O.K.?”” (page 1280). Another participant noted, “He [the dentist] tells me what he's going to do next. So, long before I can anticipate, he's already told me …” (page 1280). As can be seen from these quotes, dental patients seem to appreciate provision of explanations about the treatment plan. The dentist can easily keep them informed regarding each step of dental treatment in advance.

*Treatment Breaks and Use of “Stop” Signal*. Allowing breaks during treatment, checking patients' comfort levels, and using a previously agreed stop signal were strategies presented [12, 15, 17, 19]. The feeling of control can be increased by introducing choices including asking dental patients to raise their hands as a stop signal or to pause for a rest, requiring dentists to respond quickly to the patients' signals [44]. This strategy was appreciated as the following statements in Stalker et al.'s study [15] suggest: “If I [put] my hands across like this, or I was blinking continually that was, “stop,” you know, and I was in control” (page 1280). Another participant stated, “Most of the time [with this dentist] it's like, “You know the signals, right?” And I go “yeah.” And he'd always review the signals … “this is what you can do for yes, this is no, this is stop”” (page 1280).

However, anecdotal evidence suggests that some dentists experience difficulties with this method when patients disclose CSA. Dentists reported that these patients may have difficulty using appropriate stop signals, which may be perceived as additional stress factor given the responsibility of having to provide the stop signal. In this regard, it will be important for the dentist to be sensitive to body language and developing a sense of the patient's comfort level, assisting in the determination of when a break will be necessary.

*Providing Written Information*. Another strategy to manage patients' perceived lack of control is through providing written information [12]. This method appears to be particularly appreciated among patients with a history of CSA. “What I hear one day I won't hear the next due to dissociation when I shut down. I find it an awful help if people write things down when I am nervous, as I won't be listening” (page 303). As can be observed by the quote above, providing information in writing may assist patients, particularly those who tend to dissociate during dental treatment.

### 3.3. Avoidance (Theme 2)

Avoidance as a defence mechanism was the second major theme referred to by eight studies [15–21, 23]. Avoidance may comprise refusal to encounter activities, objects, and situations as they represent unconscious aggressive or sexual impulses or punishment for those impulses [45]. Dental patients who experienced sexual violence appeared to use avoidance behaviours as a coping mechanism to prevent any reminder of the past experiences.

#### 3.3.1. Sexual Violence and Avoidance

Often, individuals who have experienced sexual violence try to avoid any reminders of the experiences consciously or unconsciously [45–52]. Various forms of avoidance were identified as a result of experiencing sexual violence including dissociation as a form of emotional numbing, alterations in body perception, amnesia for painful memories, and multiple personality disorder [53]. Other types of avoidant behaviour included suicidal attempts, problematic substance use, and tension-reducing activities such as self-harming behaviours [53].

Although avoidance can provide a short-term relief, additional psychological problems have been reported including increased level of fear and anxiety, depressive symptoms, and PTSD [45, 52]. For instance, Wright et al. [52] found an association between the adoption of avoidance behaviour and severity of psychological problems.

#### 3.3.2. Dental Fear, Sexual Violence, and Avoidance

Frequent cancellations of the dental appointments were identified as a means of avoidance among dental patients who experienced sexual violence [15–21, 23]. The following statement quoted by a male participant in Stalker et al.'s study [15] illustrates this issue: “I put it off for about five or six times … my wife has been bugging me for a while now: “The dentist has been calling you, you've got to go now.” [I say] “OK, I'll call her back” and I do not call her back” (page 1280).

Stalker and colleagues [15] reported an association between dental fear and heightened sensitivity to dental procedures and visiting patterns. Invasion of personal space through an uninvited touch (either head and neck or arm) was found to be a main factor for avoidance behaviour [31]. It can be more difficult for individuals with a history of sexual violence to cope with reminders that may be perceived as continues victimisation. The imagination of such a feeling before visiting the dentist may increase the level of stress in these individuals and result in the cancellation of the appointment [12], which may lead to feelings of shame on behalf of patients and frustration for the dental practitioners [15]. The statement below reported by Dougall and Fiske [12] demonstrates the distress and dilemma that can be experienced: “I lost it when the nurse put her arm round me to comfort me, and she was only trying to help. I could not bear her to touch me and I could not tell her why” (page 303).

### 3.4. Embarrassment, Fear of Judgment, and Criticism (Theme 3)

**Figure 1:**
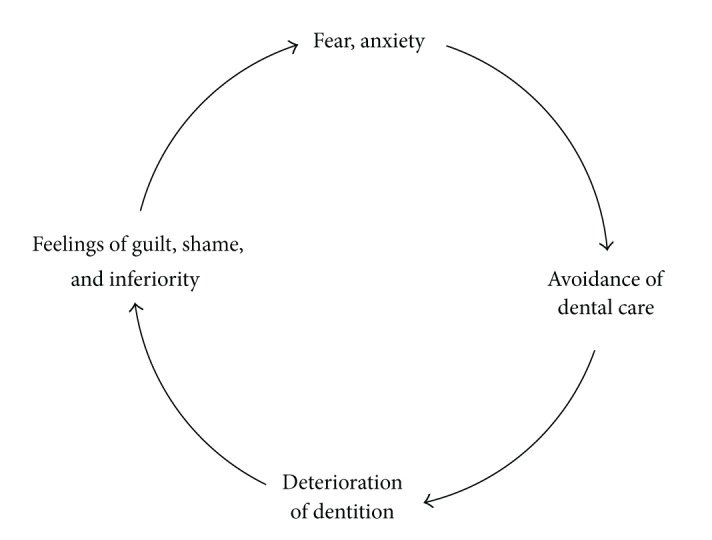
Vicious circle of dental anxiety [8, 54].

Researchers developed a “vicious circle of dental fear” framework to explain the association among avoidance, dental fear, and embarrassment. This cycle indicates how psychological variables including embarrassment in addition to dental fear result in avoidance, dental problem, and feeling of shame (Figure 1). Considering the vicious circle model, it can be observed that a high level of dental fear in patients is associated with increased avoidance behaviours resulting in extensive dental problems, which lead to feelings of guilt, shame and inferiority in the patients, and, eventually, feedback into the maintenance or exacerbation of presented dental fear [8, 54].

Four studies identified the issue of embarrassment and fear of judgment and criticism, as a prominent theme [15, 21–23], which is associated with avoidance behavior in individuals who have experienced sexual violence. For example, Stalker et al. [15] argued that feelings of shame due to fear of dental treatment among patients with a history of sexual violence prevented them from visiting the dentist. It was further reported that those patients who were constantly afraid of being reprimanded for neglecting their teeth have a tendency to avoid going to the dentist. As can be seen in the statement below, a male participant expected to be criticised for neglecting his teeth: “When they [the dentists] do my teeth, they are going to say, “Oh, you have not been taking care of them, you should have come in before”” [15, page 1280]. Additionally, experiences of childhood sexual violence by dental patients may cause a belief that they are undeserving and may expect to be harshly judged [12].

Havig [32] examined experiences of adults with a history of CSA in a health care setting. Findings suggested that exposure to sexual violence was associated with feelings of shame. This may result in neglecting personal hygiene and oral health [31]. Consequently, some individuals with a history of sexual violence victimisation may require a reminder regarding self-care and instead of reprimanding them, in fact, there is an opportunity for dental practitioners to assist sexual violence victims in maintaining a good oral hygiene [12].

#### 3.4.1. Strategies to Overcome Avoidance Behaviour

Strategies to assist dental patients to overcome the problem of frequent cancellations of dental appointment are necessary. They may include included motivation strategy and a same-day appointment strategy, which are further outlined below.

*Motivation Strategy*. Validation of dental patients' courage and energy may motivate them to maintain a good oral health. Schachter et al. [31] recommended the utilisation of simple method for improving the oral hygiene of sexual violence victims. For example, an electric toothbrush because of its novelty effect might help these patients take a better care of their teeth. It was further argued that sexual violence victims may improve their oral health and avoid experiences of shame and embarrassment, which would counteract previous hesitations of attending a dentist appointment.

*Same-Day Appointment*. Stalker and colleagues [15] promoted a same-day appointment strategy. This approach is beneficial as it allows taking advantage of a patient's readiness to attend a dentist appointment while at the same time avoiding the inconvenience and financial implications of repeated rescheduling. “If the patient feels “ready” on a particular day, he or she may call the office seeking to take a slot left open by a short-notice cancellation, if one is available, for that same day” (page 1280). This simple intervention may assist vulnerable individuals to attend to dental appointments more readily and resist the impulse of “backing out.”

### 3.5. Flashbacks (Theme 4)

An issue identified in five studies [11, 15, 16, 19, 20] was the experience of flashbacks during the dental care. A flashback, or involuntary recurrent memory, has been defined as “highly persistent memories of traumatic experiences that are activated automatically by features of the current environment and are accompanied by much reliving” [55, page 649]. Individuals with a history of sexual violence may reexperience the abusive situation in their imagination or thought processes as if it is occurring [11].

Perceived similarities between events of sexual violence and visiting the dentist may act as a trigger eliciting memories of victimisation experience [16, 19]. Studies demonstrated that individuals who experienced sexual violence intentionally avoid visiting healthcare facilities because of flashbacks, rapid emergence of fear, anxiety, grief, or anger [11, 15, 16, 19, 20]. Conditions that may elicit flashbacks in dental surgery include being placed in horizontal position, the sex of the dentist, physical proximity of the dentist, oral manipulation, and the smell of latex gloves and even aftershave.

### 3.6. Patient Body Position (Theme 5)

Five studies reported that reclining the dental chair could be experienced as particularly threatening to patients with a history of sexual violence [11, 15, 17–19]. For example, Stalker et al. [15] found that placing patients in the supine position triggered the memories of original sexual violence. Hays and Stanley [19] also noted that their participants experienced most discomfort with the placing of the dental chair in a horizontal position.

A sense of power imbalance due to the horizontal body position may result in heightened vulnerability to revictimisation and increased states of anxiety, particularly when dental practitioners are above the patients [11, 12, 15, 16, 19]. A male participant in Stalker et al.'s [15] study indicated feeling unsafe when being placed in a horizontal position in the dental chair. “You are … supine … with your head lowered … so you are really vulnerable physically, and many of us have been violated orally” (page 1280).

#### 3.6.1. Strategies for Making Supine Position Acceptable to Victims of Sexual Violence

Dental practitioners may use specific strategies to manage particularly those dental patients who experienced sexual violence and complain about the supine position. They include placing patients in semisupine position whenever possible, explaining the reasons for the supine position, covering the patients, and allowing monitoring the treatment procedures by the patients [12, 15, 31, 43]. Dougall and Fiske [12] reported that semisupine position of the patient in the dental chair with one foot on the ground appears to be tolerable and more favorable for individuals who experienced sexual violence and that patients feel safer and less distressed in this position.

Despite the benefits of semisupine position for dental patients who experienced sexual violence, it is not always possible to carry out the dental treatments in the semisupine position particularly during the procedures that need to be performed on upper jaw [12]. In such a case, providing a very detailed explanation for the patients that outlines why a supine position is required for a particular intervention to achieve the best possible quality of treatment was recommended. If patients are given detailed information why the supine position affords better visibility and, therefore, should not be avoided, they are likely to agree [12, 15].

Another strategy when the supine position cannot be avoided is the provision of cover such as blanket, coat, or X-ray bib. Schachter et al. [31] and Williams [43] suggested that this method helps the patients feel more secure and less exposed within the dental environment. As a result, they tend to experience less anxiety and panic during the procedure.

An additional experience of safety may be achieved by providing instruments that assist patients to monitor the dental procedures (e.g., intraoral camera, a mirror) [15, 43]. Such an easy strategy is likely to remove uncertainties of dental procedures [43] and allows patients to feel less vulnerable.

### 3.7. Sex of the Dentist (Theme 6)

Three studies investigated the association between the sex of the dentist and dental fear among participants with a history of sexual violence [11, 16, 19]. Hays and Stanley [19] found an association between distress symptoms and the dentist's sex. The researchers reported that the rate of nausea was significantly lower among participants who visited a female dentist. This finding was corroborated by Willumsen [16] who indicated that women with history of CSA reported male dentist to be more fear provoking.

By contrast, Leeners et al. [11] reported that participants had no preference towards the sex of their dentist. They argued that sometimes the relationship with a neglectful mother might have had a negative impact on those who experienced sexual violence, which may counteract the relationship with a perpetrator. Furthermore, given that the vast majority of perpetrators are men, there are still some individuals who are sexually victimised by women [11]. Consequently, it is fair to argue that being a male dentist may not necessarily constitute a trigger for victims of sexual violence. Patients with a history of sexual violence may feel vulnerable visiting a male or female dentist [11] and the sex of the dentist may need to be considered if there is a specific preference [12]. A patient who feels more comfortable to be treated by a female dentist should be encouraged and educated about the importance to have dental treatment by a woman as opposed to a man, without feeling guilty, given that this simple approach may prevent triggering flashbacks because of the dentist's sex.

#### 3.7.1. Strategies to Decrease the Likelihood of Revictimisation

Dougall and Fiske [12] recommended having a chaperone to help dental patients cope with the situation. The chaperone can be a member of dental team or preferably a trusted friend of the patients who can assist in coping with the distress of visiting the dentist. Another method they proposed is to leave the door of surgery open, as this may reduce the level of stress in the patients as they feel more safety.

### 3.8. Physical Proximity to the Dentist (Theme 7)

The physical closeness of the dentist, which could act as a trigger eliciting negative memories of the original act(s) of sexual violence, was mentioned by two studies [18, 19]. Hays and Stanley [19] stated that their participants experienced the most discomfort when the dentist was too close to them or touching their body. Similarly, Willumsen [18] indicated significant negative impacts from physical intimacy. Participants in both studies reported that the physical proximity to the dentist increased their level of fear and anxiety as a result of flashbacks and intrusive thoughts.

#### 3.8.1. Making the Position of the Dentist Acceptable to Victims of Sexual Violence

The dentist's body position and provision of ongoing information were found to be helpful strategies. Hays and Stanley [19] reported that the most helpful strategy was to be mindful of body position during treatment and not to lean in towards or touching the patient's body. Such considerations can improve patients' feelings of safety and prevent negative memories of victimisation events.

Providing ongoing information about the treatment was reported to be a useful strategy [31]. Informing patients in advance regarding the possible unintentional physical contacts can help dental patients who have been victimised by sexual violence to feel more secure and comfortable in the dental settings and also assist the dentist to render high quality treatments [12].

### 3.9. Oral Manipulation and Fellatio Special Effects (Theme 8)

The especially negative effect of fellatio was referred to by three studies [16, 17, 19]. These studies found an association between a history of forced oral sex and high levels of dental fear. Willumsen [16] reported that women who experienced sexual violence including fellatio have higher levels of dental fear when compared to women with sexual violence victimisation not involving fellatio. Negative impacts of forced oral sex might present a particular difficulty with oral health care in the later stages of life due to a symbolic recreation of the experience [17]. Similar findings were reported by Hays and Stanley [19]. Consequently, considering the impact of fellatio is imperative.

It is undeniable that dental procedures require the intrusion of the mouth and touching of the lips with instruments and/or the dentist's fingers. Therefore, there is a particularly high possibility that patients who experienced sexual violence involving fellatio are particularly affected [11]. This experience can result in an especially heightened state of anxiety and activation of a gag reflex preventing those patients from enjoying the benefits of dental care [12].

#### 3.9.1. Strategies to Assist Victims to Cope with the Effects of Fellatio

Dentists need to control the gag reflex among the dental patients who experienced forced oral sex [12]. A tool kit comprising several components including (a) relaxation, distraction, and desensitization techniques, (b) complementary therapies including transcutaneous electrical nerve stimulation (TENS) and hypnosis, and (c) psychological and behavioural therapies may enable dentists to choose the most appropriate strategy to providing dental treatments for patients suffering from anxiety and gag reflex [56].

Dougall and Fiske [12] strongly advised against pharmacological control therapies or chemical restraint as strategies to control the gag reflex among dental patients with a history of sexual violence involving fellatio. Such an application* per se* may result in flashbacks and subsequently heighten their state of fear and anxiety.

### 3.10. Smell of Latex Gloves and Aftershave (Theme 9)

Some victims of sexual violence may be sensitive to the smell of latex gloves. Victims felt reminded about the assault and could not tolerate the smell of latex gloves as it considerably increased the levels of fear and anxiety: “[Visiting] dentists, for me is, even on a good day, a total, absolute nightmare” [15, page 1281]. Stalker and colleagues' study [15] found that the smell of latex gloves triggered memories of the smell of a condom used in unwanted sexual encounters, which increased the patients' levels of fear.

The smell of aftershave might also trigger memories of victimisation events making the dental visit a frightening ordeal [12]. The following statement indicates that some patients with sexual violence histories behave in an uncontrolled way when they smell aftershave scent due to the smell triggering a flashback: “When the dentists smelt of that after-shave—I really freaked out” [12, page 299].

#### 3.10.1. Strategies to Address the Problem with Latex Gloves and Aftershave

Two simple but useful strategies were recommended by to help the dental patients with a history of sexual violence cope with the discussed situations, namely, the use of vinyl gloves and avoiding wearing aftershave [12, 15]. Wearing gloves is an essential part of infection control in the dental settings [57], which cannot be avoided. However, if dental patients with a history of sexual violence are anxious about the use of latex gloves, the dentist may consider the use of vinyl gloves as an alternative [15]. Dougall and Fiske [12] recommended avoiding use of aftershave in the dental surgery during the treatment of the patients because aftershave may heighten the levels of fear and discomfort experienced by patients, particularly if they have a history of sexual violence.

### 3.11. Public Awareness and Dentists' Knowledge of Association between Dental Fear and Sexual Violence (Theme 10)

Four studies [11, 15, 16, 18] referred to the general lack of public awareness and dentists' knowledge of association between dental fear and sexual violence, as a prominent theme. These studies demonstrated that women who were exposed to sexual violence might have greater difficulty in establishing a trusting relationship when compared to patients without sexual victimisation. Stalker and colleagues [15] found that the relationship between sexual violence victimisation and dental treatments along with its negative impacts on patients' current dental conditions is not clearly understood by both affected patients and their dentists. That is why only a few patients in their study informed the dentists about their previous experience of sexual violence.

Leeners et al. [11] reported that only one-third of women with a history of CSA believed that disclosing information to the dentist would be potentially useful and result in improved dental treatment. Other participants expressed that the history of CSA was not taken into account by their dentist in a serious way.

#### 3.11.1. Strategies to Enhance Dentists' Knowledge and Public Awareness

There is a need to further educate dental practitioners about dental fear and its possible association with sexual victimisation. Willumsen [18] indicated that patients with a history of sexual violence have particular difficulties establishing a trusting relationship. As a result, all members of the dental team, particularly dentists, should learn how to identify the nonverbal sign of the patients' fear and anxiety including tenseness, unusual request, and unfounded fears [12]. This analysis revealed that although dental patients with a history of sexual violence were aware that voicing their discomfort is difficult, they could speak loudly using their actions and body language [15, 17, 19], for instance, gaging when their mouths are intruded, cringing when the dentist touches them, and feeling terrified when being placed in the horizontal position. As a result, all dentists should be prepared to identify and manage such a situation.

Furthermore, it is essential for dentists to know how to ask patients about a possible history of sexual violence. Stalker and colleagues [15] stated that asking a direct question regarding sexual victimisation may result in patients' unwillingness to answer such a question honestly. Instead, they recommended asking less specific questions. For example, “Are there any parts of dental treatment that are particularly difficult for you? Is there anything we can do to help you feel more comfortable?” (page 1280). Utilising these questions by dentists will provide a situation that allows dental patients who experienced sexual violence to respond in an appropriate manner matching their level of comfort [15]. Asking these questions may also lead to the disclosure of a history of sexual violence, which can set the stage for a collaborative working relationship between dentists and dental patients who have a history of sexual violence [15].

### 3.12. Disproportionate Dental Problems

Hays and Stanley [19] investigated the association between type and incidence of dental problems and a history of sexual violence. The researchers reported that the problems with temporomandibular joint (TMJ), bruxism, gingivitis, and number of extracted teeth were significantly higher in the participants with a history of sexual violence when compared with the general population.

#### 3.12.1. Strategies to Reduce the Rate of Dental Problems of Patients Who Are Victims of Sexual Violence

No specific strategy could be located within the analysis of the included studies to address the higher incidence of oral and dental diseases in individuals who were exposed to sexual violence within this analysis. However, all strategies addressing the underlying causes of dental fear among patients, particularly those experiencing sexual violence, appear to be useful in assisting dental patients to reduce the rate of dental problems and maintain a good oral condition. For example, recommending using an electric toothbrush may assist these patients maintain a good oral hygiene [31].

## 4. Conclusion

This review examined the relationship between dental fear and a history of sexual violence victimization using a systematic review methodology. The articles analysed yielded 10 prominent themes associated with dental fear in dental patients with a history of sexual violence victimisation. Data synthesis indicated that the issue of control was the most significant theme that emerged. The most important issue for dental phobic patients seems to be their perceived lack of control and the fear of negative dental experiences. Patients with a history of sexual violence victimisation reported feeling revictimised given the fear-provoking experience of dental treatment. Consequently, patient control is a critically important issue to increase patient perception of safety in dental environments. A collaborative approach using shared control by obtaining informed consent (asking permission for dental treatment), using the “inform before you perform” slogan, allowing breaks during treatment, and using a stop signal and checking comfort levels during treatment were suggested approaches.

A number of conclusions can be drawn from this analysis. Overall, it demonstrated a clear association between dental fear and a history of sexual violence victimisation. All studies indicated that victims of sexual violence are more likely to suffer from dental fear when compared to patients without a history of sexual victimisation. It was also found that an experience of sexual violence is likely to result in the development of dental fear or at least exacerbates the presented dental fear in dental patients.

Current knowledge on oral health effects of experienced sexual violence is emerging with more or less consistent practical applications of this knowledge, particularly in the United State of America. Importantly, this study revealed that overall knowledge of the consequences of sexual violence on dental fear and oral health is only in its infancy. This became evident during the data extraction phase that only a small number of international studies investigated the association between dental fear and a history of sexual violence victimisation. Consequently, we recommend specific research in this area to generate further empirical evidence on the issues discussed. It is of concern that the current lack of knowledge and insight of some dentists may restrict patients the opportunity to receive high quality and sensitive dental treatments, particularly if they have been subjected to sexual violence. Clearly, the importance of managing these patients has not been stressed sufficiently yet and it appears imperative to improve understanding of this relationship among dental practitioners.

## Figures and Tables

**Table 1:** Terms used for literature search.

Number	Terms
1	Dental fear, dental phobia, dental anxiety, and dental phobic patients.

2	Sexual abuse, sexual violence, sexual assault, child sexual abuse, rape, incest, fellatio, and traumatic experience.

**Table 2:** Publications included in the analysis.

ID	Reference	Year	Participants number and age	Setting and location	Research design	Variable	Prevalence	Specific findings
1	[17]	1996	462 F, 18–45 y	Clinical, Seattle, USA	Mixed method	Dental fear, CSA, CPA, CN, ASA, and APA	High dental fear = 25.5%	High levels of dental fear in women are significantly associated with CSPA, CN, and ASPA.

2	[16]	2001	99 F, 17–67 y	Clinical, Norway	Quantitative	Dental fear, CSPA, and ASPA	Extreme dental fear: ST group = 23.1%, IC group = 29.3%, and OP group = 52.3%	OP during SV was found to be predictive of the development of dental fear.

3	[18]	2004	108 F, 20–60 y	Clinical, Norway	Quantitative	Dental fear, CSA	Women with CSA = 53.7%	Participants in fear and abuse group reported higher levels of dental fear in comparison with other groups. Concerning dental problems, fear and abuse and fear only group indicated more problems than the control group.

4	[19]	1997	181 F	Clinical and community, USA	Quantitative	CSA	Women with CSA = 72.9%	(i) History of CSA is associated with current women's dental experiences. (ii) Dental fear is associated with a history of sexual violence victimisation.

5	[15]	2005	58 M and 19 F, 24–62 y	Clinical, Canada	Qualitative	CSA	All participants were sexually abused	People who have experienced sexual violence often dissociate as a way of coping with overwhelming stimuli.

6	[11]	2007	255 F, mean 38.65	Clinical and community, Germany	Quantitative	CSA	Combination of SA and PA in women with CSA = 66% PA in women with CSA = 92.9%	(i) History of CSA is associated with increased dental fear. (ii) CSA experiences may increase psychological strain during dental treatment.

7	[20]	2006	240 M and F, 19–79 y	Clinical, Netherland	Quantitative	Dental anxiety, SV	HDA = 58.75%	Participants with HDA were more likely to have experienced SV as those with LDA.

8	[21]	2009	1462 M and F, 18–85 y	Community, Germany	Quantitative	Dental anxiety, SV	SV = 4.6%	The rates of high dental fear related to the SV experience among those participants were 11.9% and 4.5%, respectively.

9	[22]	2011	1024 students, 16–≥60 y	Community, UK	Quantitative	Dental anxiety, SV	HDA = 11.2%	Significant prevalence of high dental anxiety was found among participants who experienced SV.

10	[23]	2004	16 M and 14 F, 20–65 y	Clinical, Denmark	Qualitative	Dental anxiety, SV, and embarrassment	SV = 10%	Severe dental fear is associated with remarkable psychological impairment due to avoidance of routine dental checkups and treatments and subsequently deteriorating individuals' oral hygiene.

APA: adult physical abuse; ASA: adult sexual abuse; CN: child neglect; ASPA: adult sexual and physical abuse; CPA: child physical abuse; CSA: child sexual abuse; CSPA: child sexual and physical abuse; F: female; HDA: high dental anxiety; IC: intercourse; LDA: low dental anxiety; M: male; OP: oral penetration; PA: physical abuse; SV: sexual violence; ST: sexual touching.

**Table 3:** Themes identified.

Themes/study	Walker et al.'s study, 1996 [17]	Hays and Stanley's study, 1997 [19]	Willumsen's study, 2001 [16]	Willumsen's study, 2004 [18]	Moore et al.'s study, 2004 [23]	Stalker et al.'s study, 2005 [15]	De Jongh et al.'s study, 2006 [20]	Leeners et al.'s study, 2007 [24]	Oosterink et al.'s study, 2009 [21]	Humphris and King's study, 2011 [22]
Lack of control/feeling of being helpless and powerless	*√*	√	*√*	*√*	*√*	*√*	*√*	*√*	*√*	*√*

Avoidance/never go to the dentist	*√*	*√*	*√*	*√*	*√*	*√*	*√*	—	*√*	—

Lack of awareness and knowledge of relationship between SV and DF	—	—	*√*	*√*	—	*√*	—	*√*	—	—

Flashbacks	—	*√*	*√*	—	—	*√*	*√*	*√*	—	—

Embarrassment/fear of judgment and criticism	—	—	—	—	*√*	*√*	—	—	*√*	*√*

Physical proximity/threatening	—	*√*	—	*√*	—	—	—	—	—	—

Sex of dentist on SV	—	*√*	—	*√*	—	—	—	*√*	—	—

Problem with horizontal dental chair position	*√*	*√*	—	*√*	—	*√*	—	*√*	—	—

Fellatio specific impact	*√*	*√*	*√*	—	—	—	—	—	—	—

Disproportionate dental problems	—	*√*	—	—	—	—	—	—	—	—

Smell of latex	—	—	—	—	—	*√*	—	—	—	—
